# P10 - Antileukotriene treatment in children with bronchial asthma and allergic rhinitis

**DOI:** 10.1186/2045-7022-4-S1-P65

**Published:** 2014-02-28

**Authors:** Maleyka Karimova

**Affiliations:** 1 Azerbaijan Medical University, Baku, Azerbaijan

## 

In recent years, scientists around the world are paying attention to the role of leukotrienes in the pathogenesis of bronchial asthma and allergic rhinitis. Leukotrienes – a group of biologically active substances produced from the metabolism of arachidonic acid. Increasing concentrations in the blood cause bronchoconstriction, increased bronchial secretion glands, increased vascular permeability. In allergic rhinitis, after exposure to an allergen, CysLTs are released from the nasal mucosa during both phases of the allergic reaction (early and late) and manifest symptoms of allergic rhinitis. These properties determine the pronounced therapeutic effect targeted therapy antileukotriene agents.We used antileukotriene treatment (Montel) of 56 children with asthma and 34 children with allergic rhinitis. Montel (ABDI IBRAHIM, Turkey) (active ingredient montelukast) – drug, which leukotriene –receptor antagonist, significantly reduces the manifestations of asthma and allergic rhinitis, thus improving pulmonary function and nasal breathing, reduces the number of exacerbations. In addition, advantage is ease of preparation and availability of the application. Montel application in pediatric practice ensures the stability condition of children with asthma and allergic rhinitis, and to reduce the dose of hormonal drugs (inhaled steroids). Also proved to be effective prophylactic use of the drug before physical exertion in cold air, and various stress effects.Montel is available in three dosages: Montel (4 mg) for children from 2 years to 5 years and Montel (5 mg) for children from 6 years to 14 years in the form of chewable tablets. Administered orally 1 chewable tablet 1 time a day in the evening. Reception of a preparation is not dependent of the meal.Montel (10 mg, tablets, film-coated) – for adults and children over 15 years of age should take 1 tablet (10 mg) 1 every day in the evening.

